# Promotion of training course on ICD-10 Poisoning coding : necessity to adopt preventive strategies

**DOI:** 10.1186/s12909-023-04879-w

**Published:** 2023-11-27

**Authors:** Farkhondeh Asadi, Shokoofeh Afkhami, Farideh Asadi

**Affiliations:** 1 Department of Health Information Technology and Management, School of Allied Medical Sciences, Shahid Beheshti University of Medical Sciences, Darband St, Ghods Square, Tehran, Iran; 2 Head of Human Resources Training and Improvement Department, Ministry of Industry, Mine and Trade, E-Commerce Development Center, Tehran, Iran

**Keywords:** Training courses, ICD-10 coding, Coding rules, Poisoning

## Abstract

**Background:**

Poisoning is considered the most common cause of referral to emergency departments and hospitalization in the intensive care unit (ICU). Training or retraining of coders and ensuring the positive impact of these trainings in assigning accurate codes to poisoning cases is necessary to adopt practical health measures for optimal management of this disease. The present study aimed to evaluate the impact of holding a training course on poisoning coding rules based on ICD-10 in clinical coders.

**Methods:**

This study is descriptive and analytical. With the target population included the coders of hospitals affiliated with Shahid Beheshti University of Medical Sciences (*N* = 45). In order to evaluate the training course on poisoning coding rules, the Conex Input Process Product (CIPP) evaluation model was used. This model was the first goal-oriented approach evaluation model. According to the CIPP model, evaluation of the training course held in four components, including Context factors (course objectives and priority of objectives), Input factors (instructor, curriculum, facilities, equipment, and training location), Process factors (teaching process, learning, management, and support), and Product factors (feedback, knowledge, and skills). A researcher-made questionnaire containing 39 questions with a 5-point Likert scale was used to collect data. The validity of the questionnaire was calculated through content validity, and its reliability was calculated using Cronbach’s alpha coefficient (alpha = 90% in all components). In order to analyze the data, descriptive statistics (frequency percentage distribution) and inferential statistics (one-sample t-test) were used.

**Results:**

The findings of this study were presented in four components of context, input, process, and product evaluation. The average criterion for all questions in the questionnaire was considered 3. As a result, the significance level obtained from the one sample t-test was equal to *P* = 0. 0001.The training course had a favorable effect in terms of context, input, process and products.

**Conclusion:**

The knowledge and skills of clinical coders can be enhanced by updating medical knowledge, holding training courses, workshops, seminars, and conducting clinical coder accreditation. Extensive and continuous training for clinical coders is essential due to the impact of code quality on financial forecasting, electronic health records, and conducting research.

## Background

Currently, poisoning is considered the most common cause of referral to emergency departments and hospitalization in the intensive care unit [1, 2]. As many as six to eight million cases of poisoning occur annually in the United States, and roughly $ 99.1 billion is spent on treatment [3, 4]. Of these, approximately 300,000 have been hospitalized, and 30,000 have died [5]. The incidence of poisoning in 2014 is estimated at nearly four million people [5]. In Iran, poisoning is the most common cause of hospitalization and the second leading cause of death in hospitalized patients [6]. Depending on the nature, severity, and complications of acute poisoning, it requires unique methods to plan, prevent, and effectively manage [7].

Recorded medical data and related studies, often used for health services research, are valuable tools for collecting accurate data [8]. On the other hand, to create the ability to collect and analyze this data uniformly, as well as to make significant comparisons between regions or countries, it is necessary to coding the poisoning data correctly and accurately based on a standard tool. The ICD (International Classification of Diseases and health related problems), as an international standard in coding, while providing this capability, provides the basis for providing reliable reports on the types of poisoning and related measures [9].

Accurate coding requires a coding training program. In these programs, by teaching the coding rules, they have improved the coding [10, 11]. Studies show a direct relationship between the effectiveness of coding training and the reliability of codes assigned by coders to cases. In these programs, by teaching the coding rules, they have improved the coding [12].

In examining the quality of coding in Illinois hospitals in the United States, they concluded that the quality of coding in hospitals that provide continuous coding training for coders is higher than in other hospitals [13]. The results of another study on the coding error rate in Australia revealed that in the centers where the coding rules training courses were not implemented, the error rate was higher than in centers with continuous training courses for the coders [14].

In another study, the results indicated that the program of continuous training, continuous monitoring, and feedback of coding training increases the perception of coders and improves the quality of coding. Hence, continuous training of coders allows for increased interaction with clinical staff [15]. In some studies, the importance and effectiveness of coders’ professional training were emphasized through a coherent management structure and data quality improvement [16, 17]. Due to the increasing incidence of poisoning and complications, mainly in developing countries where poisoning is associated with a high mortality rate, information to coding the Poisoning play an essential role in the implementation of control programs and its complications [18, 19]. In a study, assigning accurate codes to carbon monoxide poisoning and not coding it with unknown external agents leads to targeted monitoring of this type of poisoning [20]. In addition, the use of poisoning codes using ICD classification is considered one of the criteria for measuring the prevalence of drug poisoning [21]. In South Korea, using statistics on pesticide poisoning codes using the ICD-10 classification has led to the presentation of health strategies and interventions to control this type of poisoning [22]. In a similar study, the importance of the statistics provided for accurate coding of heroin poisoning in the hospital emergency department was confirmed to monitor and control opioid poisoning [23].

### Objectives

The present study aimed to evaluate and promote the impact of holding a training course on poisoning coding rules based on ICD-10 in clinical coders and adopt preventive strategies through the promotion of training courses.

## Methods

The present study is a descriptive and analytical study with the target population included the coders of hospitals affiliated with Shahid Beheshti University of Medical Sciences (*N* = 45). In order to evaluate the training course on poisoning coding rules, the Conex Input Process Product (CIPP) evaluation model was used. This model was the first goal-oriented approach evaluation model by stufflebeam,et al. [24].

According to the CIPP model, evaluation of the training course held in four components, including Context factors (course objectives and priority of objectives), Input factors (instructor, curriculum, facilities, equipment, and training location), Process factors (teaching process, learning, management, and support), and Product factors (feedback, knowledge, and skills).

The context effectiveness of the training course, its purpose is to provide a logical context for determining educational goals, and this stage of evaluation includes analytical efforts to determine elements related to the environment and efforts to identify problems, needs, and opportunities in an educational situation. In the step of input effectiveness, the required information about how to use the resources to achieve the goals of the program is collected. In the process of input effectiveness, the nature of the ability of the educational system and potential strategies to achieve the goals identified as a result of the context effectiveness are determined. Also, at this stage, decision makers are helped to design the methods that are necessary to achieve the goals. The process effectiveness of the training course, at this stage, efforts are made to answer questions such as, is the program being implemented well? What are the obstacles to its success? What changes are necessary? In product effectiveness, the real results are determined. After the complete review of the results, the necessary information is provided to the decision makers to decide whether to continue the program or stop it if necessary.

The questionnaire with 39 questions were administered to 45 coders of the hospital, these coders who work in Health information management department of hospital, study medical records to coding diagnoses based on ICD. This questionnaire is a researcher – made with a 5-point Likert scale (strongly agree to strongly disagree) was used to collect data. The validity of the questionnaire was calculated through content validity, and its reliability was calculated using Cronbach’s alpha coefficient (alpha = 90% in all components). In order to analyze the data, descriptive statistics (frequency percentage distribution) and inferential statistics (one-sample t-test) were used. The evaluation questions of the poisoning coding training course were divided into 4 parts. The first part(context) includes 9 questions about the goals and priorities of the course, the second part (input) includes 10 questions about strategies and the educational program, the third part (process) includes 11 questions about the matching of strategies with goals, evaluation feedback, program implementation and obstacles and necessary changes to the program and department. The fourth part(products) included 9 questions about necessary reforms in conducting the training course and unforeseen cases. One time training was held and also, Pre-test and post-test were designed to evaluate the rate of poisoning coding rules and guidelines by coders.

## Results

The findings of this study were presented in four components of context, input, process, and product evaluation. According to the questions of the research questionnaire on a 5-point Likert scale (strongly agree to strongly disagree), the average criterion for all questions was considered 3. As a result, the significance level obtained from the single sample t-test was equal to *P* = 0.0001. The results of evaluating the effectiveness of the training course on poisoning coding rules in terms of context, input, process, products is presented in Table 1.

**Table 1:** Results of one-sample t-test in evaluating the effectiveness of the training course on coding rules of poisoning in terms of context, input, process, product components based on ICD-10

Mean criterion, equal to 3					
Component	Total	Mean	t	Df	P.
The context effectiveness of the training course	45	3.18	4.365	44	0.000
The input effectiveness of the training course	45	3.22	4.189	44	0.000
The process effectiveness of the training course	45	3.28	4.376	44	0.000
The product effectiveness of the training course	45	3.18	4.365	44	0.000

According to Table 1, a significant level obtained from a one-sample t-test was equal to *P* = 0.000. This means that a significant difference (*p* < 0.01) was observed between the mean of the result and the mean of the criterion. As a result, the training course has had a favorable effect in terms of context.

Regarding the input component, the significance level obtained from the t-sample’s equation was equal to *P* = 0.000. This means that a significant difference (*p* < 0.01) was observed between the mean of the result and the mean of the criterion. As a result, the training course has been highly effective in terms of input.

The findings also indicate the optimal effectiveness of the training course in terms of process. Regarding the product component, the significance level obtained from the one-sample t-test was equal to *P* = 0.000. This means that a significant difference (*p* < 0.01) was observed between the mean of the result and the mean of the criterion. As a result, the training course has had a desirable effect in terms of product.

## Discussion

Poisoning has severe and irreversible effects on human health and, if left untreated, can lead to death [3]. According one study, the causes of poisoning in individuals are diverse, and its range varies based on the extent of toxic chemical agents. Accordingly, it is necessary to adopt unique methods for collecting relevant data to plan, prevent, and manage proper and effective poisoning according to the nature, severity, and complications of acute poisoning [7]. Therefore, a standard tool is needed to create the ability to collect and analyze data uniformly and make significant comparisons between similar data to be able to conduct preventive strategies. ICD is considered a standard tool for coding and classifying this data, as well as reporting for health monitoring in the health system [25]. According to another study, the reliability of diagnosis coding is essential for quality management and care financing [26].

Healthcare staff cannot make optimal patient care decisions without high-quality coded data [27]. Also, the importance of accurate and consistent coding of diagnoses and measures was emphasized. According to the results of this study, while emphasizing the compliance of the requested codes with the type of diagnosis and treatment (to pay for services by the patient), continuous training programs to accurately coding diagnoses and measures, audit, evaluate, and monitor the competence and accuracy of coders considered an essential factor [28].

The knowledge and skills of clinical coders can be enhanced by updating medical knowledge, holding training courses, workshops, seminars, and conducting clinical coder accreditation. Extensive and continuous training for clinical coders is essential due to the impact of code quality on financial forecasting, electronic health records, and conducting research. One way to update and improve the knowledge and skills of healthcare staff, including clinical coders, is to provide in-service training. In addition, developing coding training programs for coding professionals using ICD-10 codes is crucial to improving coding quality [4].

Santos et al. mentioned the lack of continuous training for clinical coders as an organizational factor that effectively reduces the accuracy, completeness, and timeliness of coding. They pointed out that by providing training for clinical coders, the quality of the codes can be improved [15]. Dyers’ research also shows the importance of educational interventions in improving the quality of coding, so it is emphasized that holding educational programs has improved coding accuracy [11].

The results of assessing participants’ opinions on different dimensions of educational programs in the research of Yi, Piryani, and Mohan indicated that participants evaluate education programs in different dimensions, including the curriculum, instructor, teaching method, and educational facilities [29–31]. In the above study, in general, the level of self-reported perceived self-confidence of the participants increased significantly after the training workshop. In this research, the Kirkpatrick model with 4-choice Likert was used on faculty members. In the present study, the evaluation of clinical coders was based on the CIPP model with a 5-point Likert, which was observed at four levels of favorable effect. In the study of Mohan was designed on the effectiveness of the training program on biomedical waste management, the population of this study were clinical employees, like our study. Kirkpatrick’s evaluation tool included reaction evaluation and learning evaluation. Various factors under training include definition, classification of biomedical waste, classification, waste management process, etc. As a result of the evaluation, there was a significant improvement in the skill level of the respondents after completing the training program, also a large number of the studied community gained knowledge about various aspects of biomedical waste management.​Considering the diversity of educational courses in terms of content, audience, results, etc., it seems that a specific evaluation model does not have the same effectiveness for all educational courses, and it is better to use a suitable model for different levels of educational programs. And also, vvarious factors such as experienced professors, appropriate facilities and equipment, educational and research budgets, and appropriate curriculum and educational environment can affect the satisfaction of educational programs [32]. Accordingly, various aspects of program implementation should be considered in evaluating training courses.

## Conclusion

In the present study, all evaluation components of the training course, including Context factors (course objectives and priority of objectives), Input factors (instructor, curriculum, facilities, equipment, and training location), Process factors (teaching process, learning, management, and support), and Product factors (feedback, knowledge, and skills) were evaluated. The results indicated the optimal effectiveness and efficiency of the training course. Therefore, it is possible to improve the accuracy of the coders’ coding for each disease by holding continuous and regular training courses on the rules of coding diagnoses based on ICD-10.

## Data Availability

The datasets used and/or analyzed during the present study are available from the corresponding author on reasonable request.

## Abbreviations

ICU: Intensive care unit
CIPP: Conex Input Process Product
ICD: International Classification of Diseases and health related problems
